# Large-Scale Fabrication of Ultrasensitive and Uniform Surface-Enhanced Raman Scattering Substrates for the Trace Detection of Pesticides

**DOI:** 10.3390/nano8070520

**Published:** 2018-07-12

**Authors:** Jia Zhu, Guanzhou Lin, Meizhang Wu, Zhuojie Chen, Peimin Lu, Wengang Wu

**Affiliations:** 1 National Key Laboratory of Science and Technology on Micro/Nano Fabrication, Institute of Microelectronics, Peking University, Beijing 100871, China; zhujia@pku.edu.cn (J.Z.); tk968810@163.com (G.L.); geoforchinky@163.com (Z.C.); 2 College of Physics and Information Engineering, Fuzhou University, Fuzhou 350116, China; lpm@fzu.edu.cn; 3 The Affiliated High School of Peking University, Beijing 100080, China; wumeizhang2001@126.com

**Keywords:** SERS, Ag NPs, coffee ring, pesticide detection

## Abstract

Technology transfer from laboratory into practical application needs to meet the demands of economic viability and operational simplicity. This paper reports a simple and convenient strategy to fabricate large-scale and ultrasensitive surface-enhanced Raman scattering (SERS) substrates. In this strategy, no toxic chemicals or sophisticated instruments are required to fabricate the SERS substrates. On one hand, Ag nanoparticles (NPs) with relatively uniform size were synthesized using the modified Tollens method, which employs an ultra-low concentration of Ag^+^ and excessive amounts of glucose as a reducing agent. On the other hand, when a drop of the colloidal Ag NPs dries on a horizontal solid surface, the droplet becomes ropy, turns into a layered structure under gravity, and hardens. During evaporation, capillary flow was burdened by viscidity resistance from the ropy glucose solution. Thus, the coffee-ring effect is eliminated, leading to a uniform deposition of Ag NPs. With this method, flat Ag NPs-based SERS active films were formed in array-well plates defined by hole-shaped polydimethylsiloxane (PDMS) structures bonded on glass substrates, which were made for convenient detection. The strong SERS activity of these substrates allowed us to reach detection limits down to 10^−14^ M of Rhodamine 6 G and 10^−10^ M of thiram (pesticide).

## 1. Introduction

After several decades of development since it was discovered on electrochemically roughened silver in 1973 [1,2], surface-enhanced Raman scattering (SERS) has become a powerful analytical tool for applications of chemical and biological molecule detection, environmental monitoring, and food safety [3,4,5,6,7,8]. SERS is able to identify molecules through vibrational fingerprint signals and can even detect single molecules [9,10]. It is well accepted that a Raman signal can be enormously enhanced by noble metal nanostructures with sub–10 nm gaps between them, which we call ‘hot spots’ [9] Over the past decades, significant efforts in the areas of electron beam lithography [11], colloidal lithography [12], chemical synthesis [13,14,15], and self-assembly [16,17,18] have been made to develop highly active SERS substrates. All of these efforts are been focused on sufficiently high electromagnetic field enhancement, good SERS signal stability, and convenience in fabrication and manipulation. However, the above-mentioned requirements are hardly being met simultaneously. Electron beam lithography, nanoimprint lithography, and colloidal lithography can fabricate highly-uniform Ag or Au nanostructures leading to stable and reproducible SERS signals, but these methods are generally expensive and time consuming for large-scale fabrication. Ag or Au nanoparticles (NPs) formed by chemical synthesis is a very popular approach to SERS substrate preparation, and tremendous Raman enhancement could be achieved effortlessly by rich ‘hot spots’. However, the downside of this kind of method is that the stability cannot be guaranteed because of the uneven distribution of ‘hot spots’. For example, the NPs often aggregate in solution, which is not conducive to long-term preservation [19]. With regard to the SERS measurement, a general and simple method is mixing analytes with NP solutions, and then measuring the mixtures directly [20]. However, this method is incapable of trace detection. An improved method is to dry the mixtures. It is true that the NPs will be closely packed after drying [21,22,23,24], but this gives rise to a challenge of terrible aggregation caused by the coffee ring effect, also resulting in signal instability. Although, a coating method has been proposed to realize uniform and high-density Ag NPs distribution in drying process [25], this method suffers from oxidation of Ag NPs as time goes on. Therefore, it is still a great challenge to fabricate large-scale SERS substrates with uniform and high-density hot spots via simple and low-cost strategies.

The coffee-ring is a pattern left by a puddle of a particle-laden liquid after evaporation, which is almost familiar to everyone [26]. It is difficult to eliminate this ubiquitous effect from many applications, including the printing, assembly, and distribution of nano/molecular materials [27,28]. Closely packing Ag or Au NPs is the easiest way to obtain SERS substrates that might have a substantial enhancement of detection signals. The coffee-ring effect will make the Ag or Au NPs form as a ring, so that the distribution of the ‘hot spots’ is nonuniform and uncontrollable [22,23,29].

Herein, we present a convenient and inexpensive strategy to fabricate large-scale SERS substrates with stable and ultrasensitive performance. It involves a green chemistry synthesis method of Ag NPs and a facile approach of dropping the Ag NPs/glucose solution to form a flat film array for SERS detection. Viscous forces from the ropy glucose suppresses the coffee-ring effect, and thus leads to a uniform and compact deposition, but not aggregation of Ag NPs. Due to the wettability of the Ag NPs/glucose film, uniform distribution of analytes is also realized. These make the SERS signal more consistent and sensitive. In this strategy, no toxic chemicals or sophisticated instruments are required to fabricate the SERS substrate. In addition, thanks to the protection of glucose, oxidation of the Ag NPs is avoided, which results in their long-term storage (at least 6 months). Finally, we demonstrate the application of such SERS substrates for detection of R6G (Rhodamine 6G) and thiram (pesticide) down to 10^−14^ M and 10^−10^ M, respectively.

## 2. Materials and Methods 

### 2.1. Materials

Silver nitrate (99.9%), R6G (C_28_H_31_N_2_O_3_Cl, 99%) and thiram (C_6_H_12_N_2_S_2_, 99.9%) were purchased from Sigma-Aldrich (Darmstdt, Garmany), Ammonia (25% *w*/*w* aqueous solution) and D-glucose were supplied by Beijing Chemical Works (Beijing, China), and SYLGARD 184 Silicone Elastomer Base and SYLGARD 184 Silicone Elastomer Curing Agent were purchased from Dow Corning Corporation (Midland, MI, USA). All the reagents used in this work were of analytical grade. Deionized water (Milli-Q purification system, Millipore Co., Bedford, MA, USA) was used for all experiments.

### 2.2. Characterizations

UV-visible spectra were recorded with a 1 cm path length quartz cell using an Agilent Cary 8454 spectroscopy system (Agilent Technologies Inc., Santa Clara, CA, USA). Polydimethylsiloxane (PDMS) surfaces were treated with a BD-20AC laboratory corona treater (Electro-Technic Products Inc., Chicago, IL, USA). Scanning transmission electron microscope (STEM) measurements were conducted on Tecnai G2 F20 (FEI, Hillsboro, OR, USA). The Raman spectra were obtained using a Renishaw inVia Reflex Raman Microscope and Spectrometer (inVia Reflex, Gloucestershire, UK) equipped with a 633 nm laser and 50× objective. The integration time of all spectra acquisition for each measurement was set to be 10 s. The laser power was 1.7 mW. Four spots on the same SERS substrate were examined, and the spectra were averaged for final analysis.

### 2.3. Preparation of Ag NPs

The Ag NPs were prepared according to our previous reports [30]. Briefly, approximately 1 mL of ammonium hydroxide was added drop by drop into a fast stirring silver nitrate solution (85 mg in 10 mL water). A dark-brownish precipitate was formed and then dissolved as the amount of ammonium hydroxide is increased. Subsequently, a small amount of as-prepared Tollens solution (40 μL) was added into a fresh solution (40 mL) of D-glucose with a concentration of 0.1 M to 0.5 M. After four hours at room temperature, Ag^+^ ions were reduced by glucose in the presence of ammonia, and the color of the glucose solution turned to yellow, indicating the formation of Ag NPs [31].

### 2.4. Fabrication of SERS Substrates

**Figure 1:**
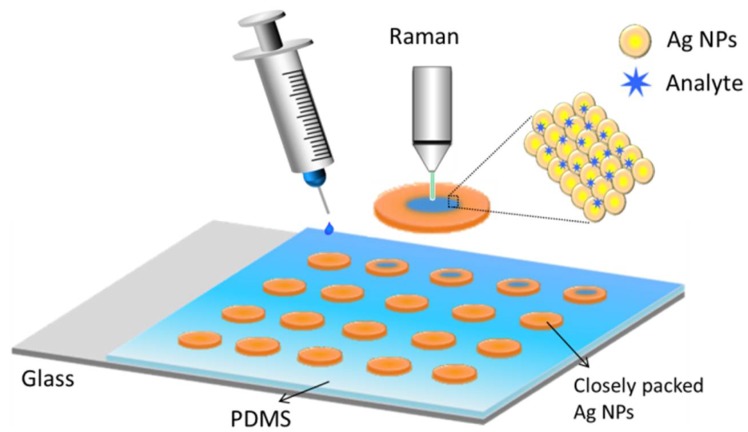
Schematics of the SERS detection process. The Ag NPs are closely packed and surrounded by massive solid-state glucose. Moreover, analytes can be distributed uniformly in the SERS substrates by infiltration and capillarity.

Firstly, the thin PDMS films with a thickness of 0.5 mm were prepared by mixing a Slygard 184 elastomer with a curing agent in a 10:1 ratio at 70 °C for two hours. Then the PDMS films were punched to form an array of holes with a diameter of 5 mm. Subsequently, the punched PDMS films were treated by O_2_ plasma using a BD-20AC laboratory corona treater (Electro-Technic Products Inc., Chicago, IL, USA) for 30 s. Then, the treated PDMS surface was bonded to a glass slide under 70 °C for 2 h. Finally, 100 μL of prepared colloidal Ag NPs were dropped into each hole of the PDMS films. After the droplets were evaporated, the pie-shaped substrates of Ag NPs were formed, and then stored at room temperature for SERS detecting. The fabricated SERS film array and the SERS detection process is shown in the Figure 1.

## 3. Results and Discussion

### 3.1. Characterization of Ag NPs

**Figure 2:**
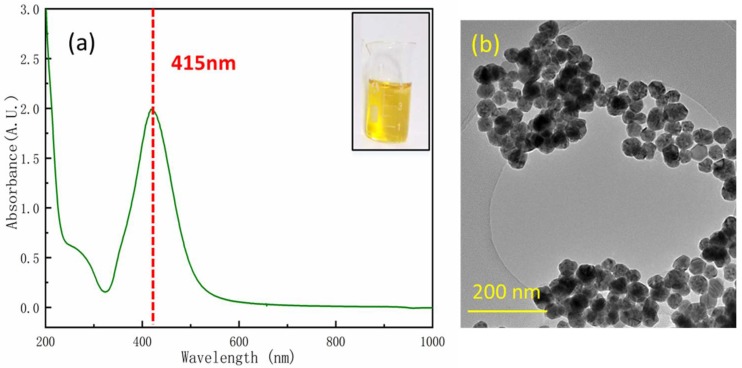
(**a**) UV-vis absorption spectra of the Ag NPs, and the inset shows a photograph of the Ag NP colloid, and (**b**) TEM image of the Ag NPs, and the size of Ag NPs were 35 ± 3 nm.

UV-vis absorption spectra of the colloidal Ag NPs synthesized in 0.5 M glucose is given in Figure 2a. The absorption peak was around 415 nm, exhibiting a sharp plasmon absorption maximum. It indicated that the Ag NPs were monodispersed and relatively uniform in a stable colloidal solution [30,31]. A TEM image of Ag NPs is given in Figure 2b. The size of the Ag NPs was 35 ± 3 nm (the UV-vis absorption spectra and TEM images of the Ag NPs synthesized in 0.1–0.5 M glucose are given in Figures S1 and S2). Since the amount of glucose is excessive, the Ag^+^ ions have been completely reduced. Moreover, it was a green and highly-efficient synthesis method that does not need a heating condition or any relatively toxic organic surfactants.

### 3.2. Suppression of the Coffee-Ring Effect/Pie-Shaped SERS Substrates

**Figure 3:**
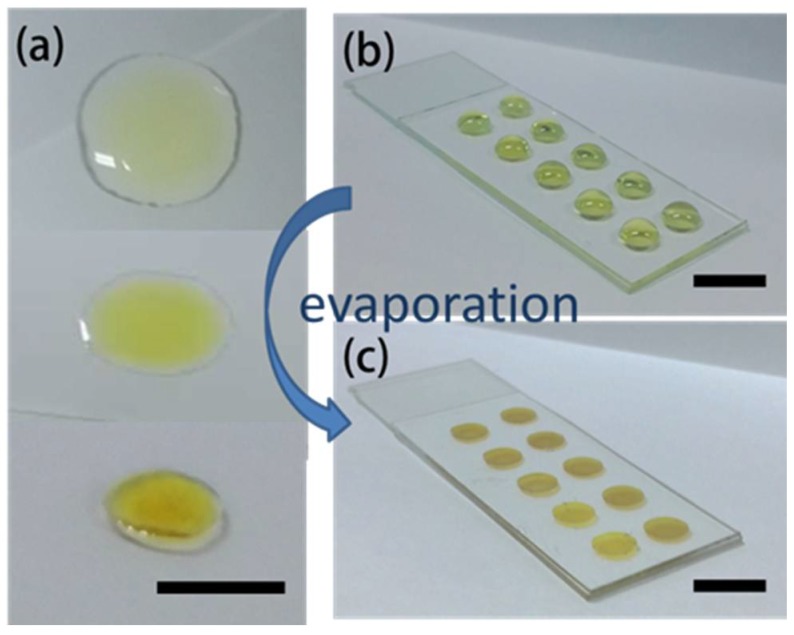
(**a**) The evaporation process of a drop of the solution, and (**b**) a photograph of colloidal Ag NPs dropped into an array of holes. The holes were 5 mm in diameter, 1 mm in depth, and 10 mm apart for standardized fabrication and convenient measurement of the SERS films in array. (**c**) The fabricated pie-shaped SERS substrates after evaporation. The scale bar is 1 cm.

When a drop of liquid dries on a solid surface, the liquid evaporates first from the edge and is replenished by liquid from the interior, which results in capillary flow in the drop during its drying process. The suspended particles are driven to the edge by the capillary flow, and then left highly concentrated along the original drop edge, finally depositing in a ring-like pattern after evaporation [28]. Many attempts to suppress and ameliorate the coffee ring effect have thus far focused on manipulating the capillary flow [32,33,34,35]. In our strategy, the solution became viscous owing to the existence of massive glucose during the evaporation of the as-prepared colloidal Ag NPs, and the capillary flow is therefore burdened by viscidity resistance, which prevented the suspended particles from reaching the drop edge and ensured their uniform deposition. Figure 3a shows the evaporation process of a drop of as-prepared colloidal Ag NPs dropped onto a clear glass directly. As the water evaporates, the concentration of the glucose is increased, which resulted in an increasing viscosity of the solution [36]. Finally, the droplet had turned into a rigid film and the coffee-ring effect was almost eliminated after evaporation. As the droplet turned to a rigid film, the volume shrunk dramatically, therefore the gap between the Ag NPs would decrease and even turn into an aggregate, which exhibits higher SERS activity [37]. In order to make the rigid film well-shaped and standardize the fabricating process, the colloidal Ag NPs were dropped into an array of holes, which were defined by PDMS structures bonded on the glass substrates (Figure 3b). Figure S3 shows the extinction spectrum of the SERS substrate with 0.2 M glucose. Thanks to the protection of glucose, oxidation of the Ag NPs was also prevented, which resulted in superior stability of the SERS substrates. From the test, the stability and sensitivity of SERS signals could remain for at least 6 months (Figures S4 and S5). The characteristic peaks of R6G were still very distinct after 6 months, and the intensity of the peaks was comparable to the intensity when the substrate was deposited.

### 3.3. Optimal Concentration of Glucose

**Figure 4:**
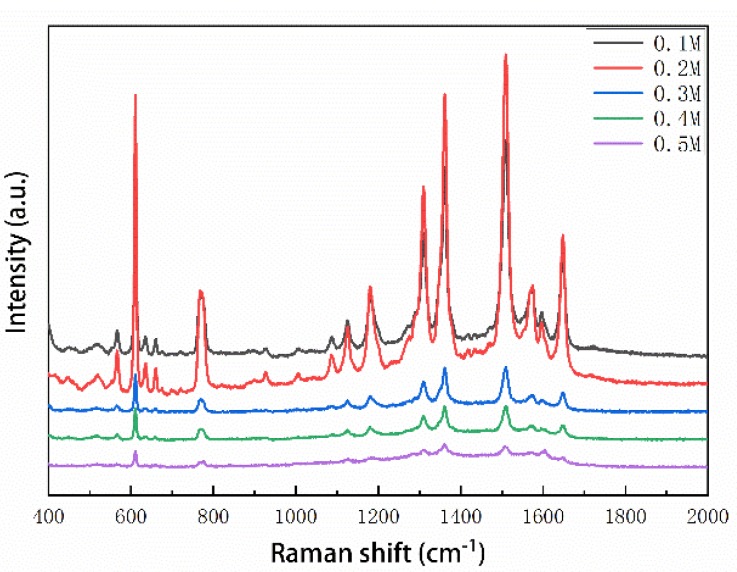
SERS spectra of 10^−8^ M R6G as different concentrations of glucose are used to fabricate the SERS substrates.

According to the enhancement mechanism of SERS signals, more sensitive SERS signal could be realized when analytes are close enough to the Ag NPs surface. In our strategy, the glucose protected the Ag NPs from oxidation while preventing the touch between the analyte and the Ag NPs to some extent. Therefore, the optimal concentration of glucose is of great necessity. In experiments, colloidal Ag NPs with different concentrations of glucose, ranging from 0.1 M to 0.5 M, were used to fabricate the SERS substrates. R6G (10^−8^ M) was employed as a SERS probe to evaluate the performance of these SERS substrates. As shown in Figure 4, the SERS signal intensity of these five substrates was in the order: 0.2 M > 0.1 M > 0.3 M > 0.4 M > 0.5 M, suggesting the substrate fabricated with a 0.2 M concentration of glucose showed a stronger signal than the others. When 1 μL of R6G solution was dropped onto the SERS substrates, the water would dissolve the glucose that covered the Ag NPs, and then the Ag NPs were exposed to analytes. Hence, a lower concentration of glucose was more likely to supply bare Ag NPs, resulting in a higher Raman enhancement. As shown in Figure S6, there was a risk of oxidation of Ag NPs with a much lower concentration of glucose (such as 0.1 M). Accordingly, we choose a 0.2 M Ag NPs/glucose solution to fabricate the SERS substrates for later detection.

### 3.4. Sensitivity and Reproducibility of SERS Substrates

**Figure 5:**
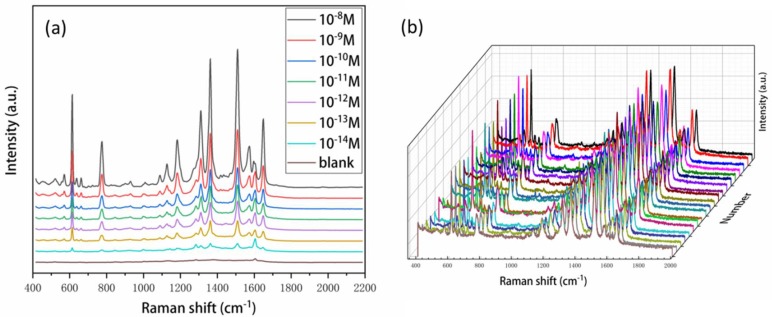
(**a**) SERS spectra of R6G at a concentration of 10^−8^ M to10^−14^ M. 1 μL of the R6G solution was dropped to the SERS substrates for detection. The blank is a control group which used deionized water. (**b**) Raman spectra of 10^−10^ M R6G collected from 20 random spots. The RSD of the intensity maximum at the peak of 1509 cm^−1^ was 6.8%.

Sensitivity and reproducibility are major concerns for any SERS substrates. Hence, we employed different concentrations of R6G to investigate the performance of the SERS substrates. As shown in Figure 5a, the strong peaks at 612 cm^−1^, 774 cm^−1^, 1127 cm^−1^, 1185 cm^−1^, 1310 cm^−1^, 1360 cm^−1^, 1509 cm^−1^, 1573 cm^−1^, and 1650 cm^−1^ were in good agreement with previous reports on pure R6G [38,39,40]. These SERS spectral feature peaks can still be clearly identified even when the concentration was down to 10^−14^ M, corresponding to 10 zeptomoles of R6G molecules in a 1 μL sample volume, which is far below previous works [38,39,40,41]. The reproducibility of the SERS substrate was further investigated by taking SERS spectra of R6G at the concentration of 10^−10^ M from 20 random locations on a single pie-shaped substrate. The average relative standard deviation (RSD) of the intensities (at 1509 cm^−1^) was 6.8% (Figure 5b and Figure S7) which is lower than previously reported [40,42], indicating that the substrates possess good signal uniformity.

### 3.5. Application for Thiram Detection

**Figure 6:**
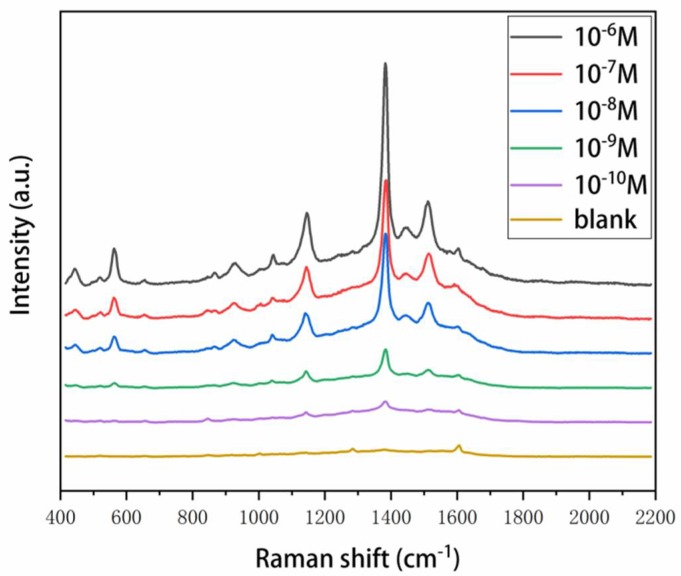
SERS spectra of thiram at a concentration range from 10^−6^ M to 10^−10^ M. The blank is a control group with DI water.

Thiram is a typical sulfur-containing pesticide molecule, which is widely used in agriculture. In this contribution, the SERS substrates were employed to detect thiram for future practical applications. The SERS spectra of thiram with a concentration varying from 10^−6^ M to 10^−10^ M are shown in Figure 6. The SERS signal intensity gradually increased with the increase of thiram concentration, pointing to the possibility of quantitative analyte determination. The main Raman bands include 563 cm^−1^ attributed to υ(S-S), 1147 cm^−1^ corresponding to ρ(CH_3_) and υ(C-N), and 1383 cm^−1^ corresponding to $\delta_{s}$(CH_3_) and υ(C-N), and 1511 cm^−1^ corresponding to υ(C-N), δ(CH_3_), and ρ(CH_3_) [42,43]. We could identify the spectrum of the thiram even at the concentration of 10^−10^ M. The result suggested that the ability of SERS substrates to have a detection sensitivity of 10^−10^ M of thiram, which is far below the maximum residue limits of thiram for vegetables (5 mg/kg, equal to ≈2 × 10^−5^ M). It is evident that such SERS substrates would have great potential for real world applications.

## 4. Conclusions

In summary, we have demonstrated a method for a simple and economically viable design of large-scale, highly efficient, ultrasensitive, uniform, and low-cost SERS substrates, especially emphasizing the suppression of the coffee-ring effect. In this method, the glucose acts to achieve this suppression. First, Ag NPs were synthesized by a modified Tollens method in which glucose serves as a reducing agent. Secondly, a Ag NPs/glucose solution was dropped into a hole-shaped PDMS structure. After evaporation, a flat and uniform SERS film array was formed by means of viscidity resistance in a ropy glucose solution, which prevented the suspended particles from reaching the drop edge and ensured a uniform deposition. Thirdly, owing to the protection of glucose, oxidation of the Ag NPs was also avoided, which resulted in long-term storage (at least 6 months) of the SERS substrates. Subsequently, the performance of the SERS substrates fabricated with different concentrations of glucose was investigated, and the result suggested that the optimal concentration of glucose was 0.2 M. Finally, we demonstrated the application of such SERS substrates for detection of R6G and thiram down to 10^−14^ M and 10^−10^ M, respectively. Thus, such a convenient fabrication method and superior performance of the obtained SERS substrates would provide an opportunity to bring the SERS technology closer to real-world applications.

## Supplementary Materials

The following are available online at http://www.mdpi.com/2079-4991/8/7/520/s1, Figure S1: UV-vis absorption spectra of the colloidal Ag NPs synthesized in 0.1–0.5 M glucose; Figure S2: TEM images of the Ag NPs synthesized in 0.1–0.5 M glucose; Figure S3: SERS intensity of 10^−10^ M R6G collected at 1509 cm^−1^ from 20 random spots of a pie-shaped SERS substrate; Figure S4: SERS spectra of R6G (10^−10^ M) collected from the SERS substrates at different storage times ((a) 0 months, (b) 2 months, (c) 4 months, and (d) 6 months).
