# Supplementary material for: Large-Scale Fabrication of Ultrasensitive and Uniform Surface-Enhanced Raman Scattering Substrates for the Trace Detection of Pesticides
Source: Nanomaterials (Basel). 2018 Jul 12;8(7):520. doi: 10.3390/nano8070520 (PMC6071232; doi:10.3390/nano8070520)
Supplement: Supplementary file 1 [file nanomaterials-08-00520-s001.pdf]

Supporting Information for publication

Large-Scale Fabrication of Ultrasensitive and Uniform Surface-Enhanced Raman Scattering Substrates for the Trace Detection of Pesticides

Jia Zhu [1], Guanzhou Lin [1,2], Meizhang Wu[3], Zhuojie Chen [1], Peimin Lu [2] and Wengang Wu [1,*]

[1] National Key Laboratory of Science and Technology on Micro/Nano Fabrication, Institute of Microelectronics, Peking University, Beijing 100871, China; zhujia@pku.edu.cn (J.Z.); tk968810@163.com (G.L.); geoforchinky@163.com (Z.C.)

[2] College of Physics and Information Engineering, Fuzhou University, Fuzhou 350116, China; lpm@fzu.edu.cn

[3] The Affiliated High School of Peking University, Beijing 100080, China; wumeizhang2001@126.com

Corresponding author:

Prof. Wengang Wu

*Email address: wuwg@pku.edu.cn

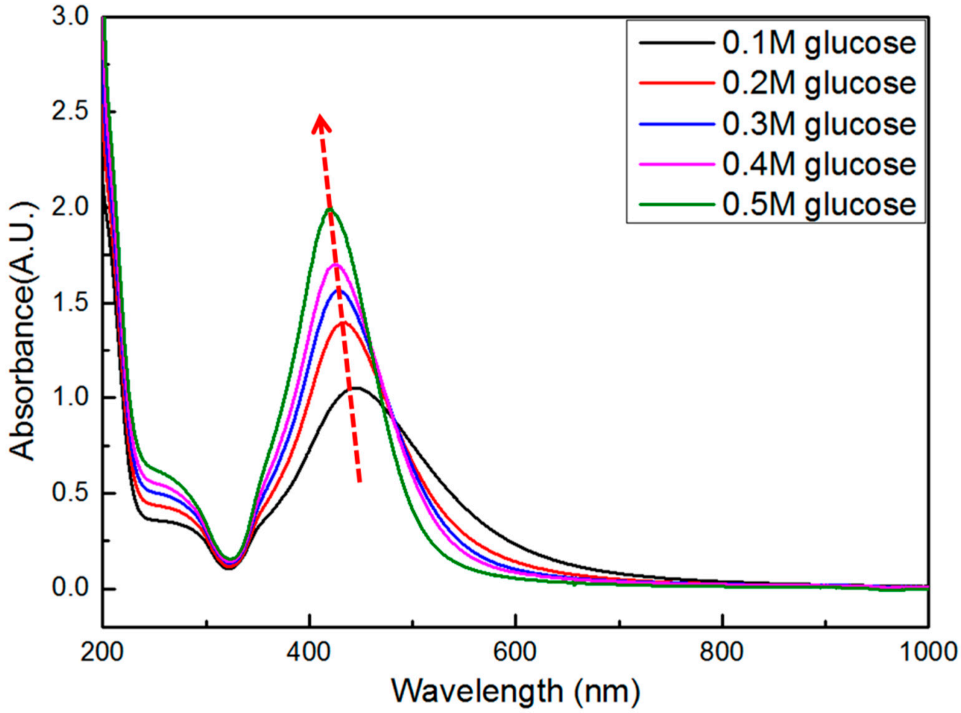

Figure S1. UV-vis absorption spectra of the colloidal Ag NPs synthesized in 0.1–0.5 M glucose. The size of the Ag NPs increased with the decrease of the concentration of glucose.

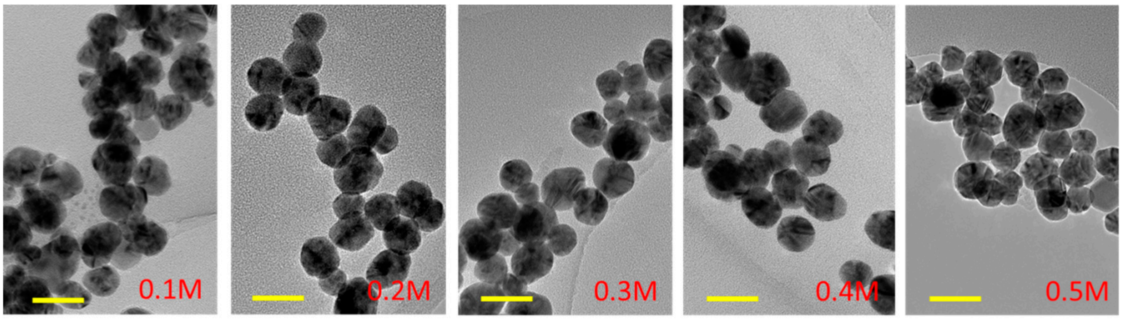

Figure S2. TEM images of the Ag NPs synthesized in 0.1–0.5 M glucose. The scale bar is 50 nm.

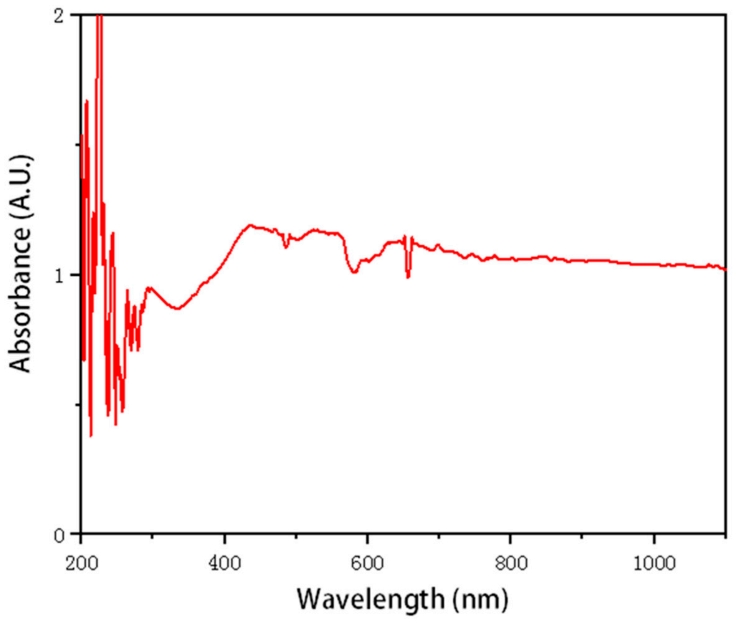

Figure S3. Extinction spectrum of the SERS substrate with 0.2 M glucose.

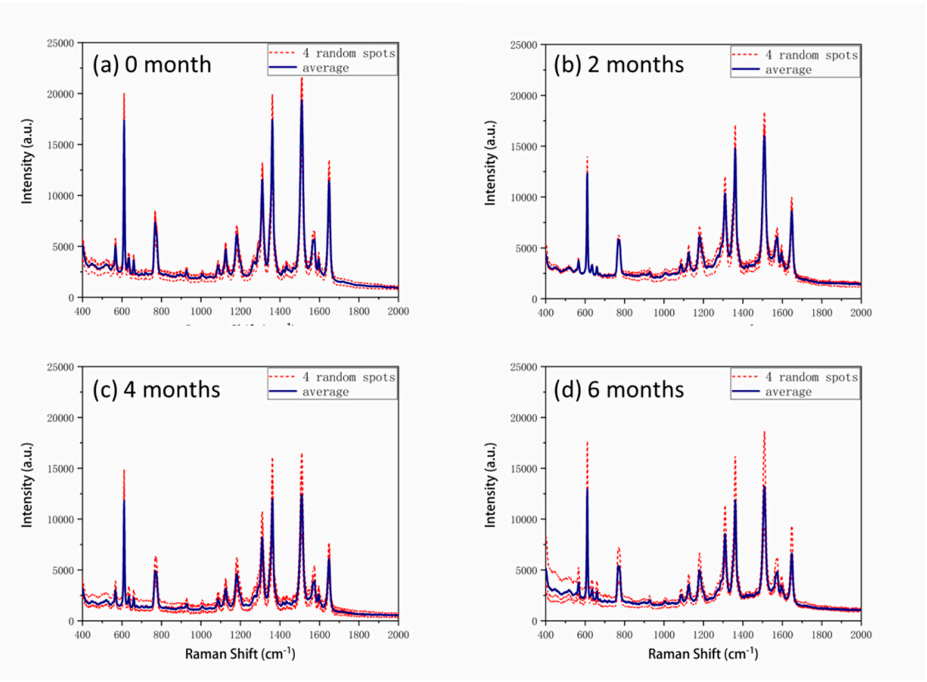

Figure S4. SERS spectra of R6G (10^{-10} M) collected from the SERS substrates at different storage times ((**a**) 0 month, (**b**) 2 months, (**c**) 4 months, (**d**) 6 months). The blue solid line is the average of four red dotted lines that were collected from four random spots on the same SERS substrate. The SERS sensitivity and uniformity of the SERS substrates kept well after 6 months, which indicated that the SERS substrates can be stored for more than 6 months.

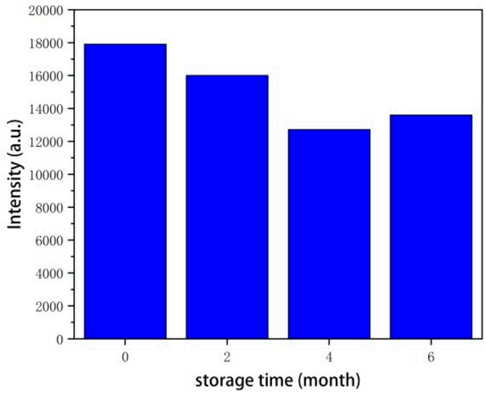

Figure S5. The change in the peak intensity (1509 cm^{-1}) at the different storage times of 0 month, 2 months, 4 months, and 6 months.

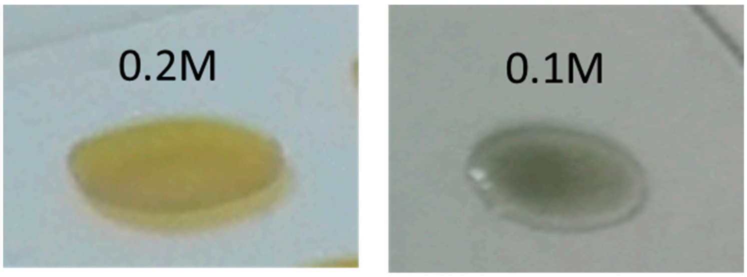

Figure S6. Photograph of pie-shaped SERS substrates with 0.2 M and 0.1 M glucose, respectively. The SERS substrate turned to black, indicating that the Ag NPs are easy to oxidize with 0.1 M glucose.

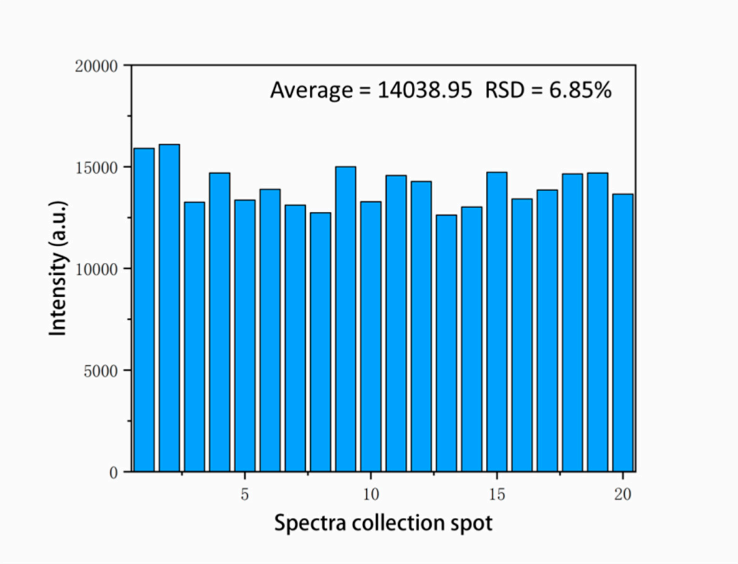

Figure S7. SERS intensity of 10^{-10} M R6G collected at 1509 cm^{-1} from 20 random spots of a pie-shaped SERS substrate. The average SERS intensity and relative standard deviation (RSD) are also calculated.
